# Exclusive breastfeeding after breast cancer. Case study*

**DOI:** 10.17533/udea.iee.v41n1e05

**Published:** 2023-03-14

**Authors:** Maria Antónia Poupas Martins, Margarida Sim-Sim

**Affiliations:** 1 RN, M.Sc. USF Planície, ARS Alentejo, Évora. Universidade Católica Portuguesa. Lisbon, Portugal. Email: mantonia.martins@alentejocentral.min-saude.pt. Universidade Católica Portuguesa Universidade Católica Portuguesa Lisbon Portugal mantonia.martins@alentejocentral.min-saude.pt.; 2 RN, Ph.D. Comprehensive Health Research Centre (CHRC). Universidade de Évora, Nursing Department. Évora, Portugal. Email: msimsim@uevora.pt Universidade de Évora Comprehensive Health Research Centre (CHRC) Universidade de Évora Nursing Department Portugal msimsim@uevora.pt

**Keywords:** breastfeeding, breast cancer, standardized nursing terminology, postpartum period, perinatal care, lactancia materna, neoplasias de la mama, terminología normalizada de enfermería, periodo posparto, atención perinatal, aleitamento materno, neoplasias da mama, terminologia padronizada em enfermagem, período pós-parto, assistência perinatal

## Abstract

**Objective.:**

To develop a care plan focused on assisting a puerperal woman who decides to feed their child through breastfeeding on the contralateral breast.

**Methods.:**

A case study conducted with a 36-year-old primiparous woman who underwent tumorectomy and left axillary emptying, chemotherapy and radiotherapy 8 years ago. The data were collected in the Nursing process, with initial assessment using the Marjory Gordon functional health patterns. The care plan used the NANDA-I, NIC and NOC taxonomies.

**Results.:**

Three diagnoses were identified: [00208] Readiness for Enhanced Childbearing Process, [00106] Readiness for Enhanced Breastfeeding and [00167] Readiness for Enhanced Self-Concept. The indicators were evaluated in the initial and later phase, with gains in the first two diagnoses. The third diagnosis proved to be partially sufficient, as it did not allow assessing the developmental and non-pathological self-image in the puerperium. In the puerperal phase, maternal roles are challenging and demanding. Nursing care contributed to the adaptation to breastfeeding in the contralateral breast and to the guarantee of adequate nutrition for the newborn. The childbearing process was strengthened. Breastfeeding is carried out with maternal satisfaction, good latch and normal weight evolution in the newborn.

**Conclusion.:**

The case study strengthened knowledge by addressing a little investigated theme. The NANDA-I taxonomy may need further study in the Self-perception domain, more specifically in the self-image reported during the pregnancy-puerperal phase.

## Resumen

**Objetivo.:**

Presentar un estudio de caso y elaborar un plan de cuidados para asistir a la puérpera que decide amamantar con la mama contralateral.

**Métodos.:**

Estudio de caso de una primípara de 36 años, a quien se le realizó ablación de tumor y disección axilar izquierda, quimioterapia y radioterapia hace 8 años. Los datos se recolectaron en el Proceso de Enfermería y en la entrevista, con evaluación inicial en la que se emplearon los estándares funcionales de salud de Marjory Gordon. El plan de cuidados utilizó las taxonomías NANDA-I, NIC y NOC.

**Resultados.:**

Se identificaron tres diagnósticos: [00208] Voluntad de mejorar el proceso perinatológico después del nacimiento, [00106] Voluntad de mejorar la lactancia y [00167] Voluntad de mejorar el autoconcepto. Después de evaluar los indicadores en la fase inicial y posteriormente, se observaron beneficios en los dos primeros diagnósticos. El tercer diagnóstico resultó ser parcialmente suficiente, ya que no permitió evaluar la autoimagen evolutiva no patológica durante el puerperio. En la fase puerperal, los roles maternos son desafiantes y exigentes. Los cuidados de Enfermería contribuyeron para la adaptación a la lactancia materna en la mama contralateral y para asegurar una adecuada nutrición del recién nacido. Se fortaleció el proceso perinatal. La lactancia materna se realiza con satisfacción materna, buen agarre y desarrollo normal del peso del recién nacido.

**Conclusión.:**

El estudio de caso benefició el conocimiento al abordar un tema poco investigado. La taxonomía NANDA-I quizás necesite más estudio en el dominio de la Autopercepción, más específicamente en la autoimagen relacionada con la fase de embarazo-puerperio.

**Objetivo.:**

Apresentar um estudo de caso e elaborar um plano de cuidados de assistência à puérpera que se decide pelo aleitamento na mama contra-lateral.

**Métodos.:**

Estudo de caso referente a primípara de 36 anos, submetida a tumorectomia e esvaziamento axilar esquerdo, quimioterapia e radioterapia há 8 anos. Dados recolhidos no processo de enfermagem e em entrevista, com apreciação inicial utilizando-se os padrões funcionais de saúde de Marjory Gordon. O plano de cuidados utilizou a taxonomia NANDA-I, NIC e NOC.

**Resultados.:**

Identificaram-se três diagnósticos: [00208] Disposição para processo perinatológico melhorado após o nascimento, [00106] Disposição para amamentação melhorada e [00167] Disposição para autoconceito melhorado. Avaliados os indicadores em fase inicial e posteriormente, nos dois primeiros diagnósticos, constataram-se ganhos. O terceiro diagnóstico revelou-se parcialmente suficiente, pois não permitiu a avaliação da autoimagem desenvolvimental, não patológica, no puerpério. Os papéis maternos, em fase puerperal, são desafiadores e exigentes. A assistência de enfermagem contribuiu para a adaptação à amamentação na mama contra-lateral e para a garantia de nutrição adequada do recém-nascido. O processo perinatalógico ficou robustecido. A amamentação realiza-se com satisfação materna, boa pega e evolução ponderal normal do recém-nascido.

**Conclusão.:**

O estudo de caso beneficiou o conhecimento, ao abordar temática pouco investigada. A taxonomia NANDA-I necessita porventura de maior aprofundamento no domínio da autoperceção, mais especificamente na autoimagem reportada à fase gravídico-puerperal.

## Introduction

Birth of a child is accompanied by unique experiences in the bio-psycho-social aspects of a woman. Puerperal women experience a challenging phase at a moment called post-natal period, puerperium or postpartum.^1^^,^^2^ Referring to the period from birth to six week of life, the World Health Organization (WHO)^3^ recommended the term “post-natal period” for the issues regarding both mothers and newborns. The WHO defines the following: a) initial post-natal period for the first 24 hours, b) early post-natal period for between 2 and 7days, and c) late post-natal period for between 8 and 42 days.^2^ In the postnatal period, the dyad is interdependent in terms of the care measures, including lactation *versus* feeding of the newborn (NB). In this way, the ancestrality that defines us in the *Mammalia* Class is fulfilled, when the option is Exclusive Breastfeeding (EBF). The WHO recommends that EBF should be practices during the first six months of life (for example, in the 54^th^ World Health Assembly; A54/INF.DOC./4; Global strategy for infant and young child feeding; May 2001). However, EBF in the first weeks of life is one of the most difficult puerperal experiences and it is not uncommon for the belief that it is insufficient to arise, thus triggering early weaning.^4^^,^^5^

In addition to the immediate benefits, EBF offers long-term gains and reduces the risk of diseases, namely breast cancer. Several theories invoke lower risk, based on the reduction of breast oestrogens, excretion of fat-soluble carcinogens,^6^ differentiation of breast tissue and reduction of the number of ovulations in the years of life.^7^ A number of studies conducted with Afro-Americans that displayed low adherence to breastfeeding showed high breast cancer rates. On the other hand, among women who breastfeed, the relative risk of breast cancer decreases in the pre- and post-menopausal periods.^8^ In some female breast cancer survivors, the motivation to breastfeed is important; however, the face problems due the need for more information and support, in addition to more sensitivity towards the obstacles.^9^ In regions such as Alentejo, where the breast cancer rates are high,^10^ the needs of women with a cancer history when they decide to breastfeed are unknown. Such fact lacks research and seeks to overcome knowledge gaps, given the recommendations of authors who demand greater investment.^11^ The objectives of this study are to present a case study and to develop a care plan for **a** puerperal woman who decides to breastfeed on the contralateral breast.

## Methods

A single-case study that illustrates a real situation. It corresponds to level of evidence 5.^12^ The Case REport (CARE) guidelines^13^ and the case study conceptualization are respected.^14^ It addresses the case of a breastfeeding puerperal woman who, on the blood sample collection day for the PKU-TSH test, requested an appointment for the 10^th^ postpartum day, performed by a Nurse Specialized in Maternal and Obstetric Health Nursing, for guidance on breastfeeding. In the unpredictability of some deterioration and considering the puerperal woman's oncological history, it was decided to immediately assess the case, in the light of Marjorie Gordon's Functional Health Standards, not observing any critical change (Table 1). 

**Table 1:** Assessment according to Marjorie Gordon's Functional Patterns

Patterns	Analysis
Health perception and management	She perceives physiological changes during the post-natal period; she invests in managing the puerperal changes, maintaining high puerperal surveillance due to the high oncological risk. There is a need to better manage some physiological changes [lactation process, engorgement prevention] that are reasons to schedule an EESMO appointment.
Nutrition and metabolism	Nutrition and metabolism suggest puerperal normality. Nutrition of the NB is a cause for concern, as EBF is only performed on the contralateral breast. In the Nursing evaluation, explanations are given and the problem is reduced, due to the NB's adequate status-weight development.
Eliminations	Normal bladder and intestinal eliminations. Right breast: excretion of transitional milk [1^st^ PHC visit], at the time of Early Diagnosis to the NB (foot test). Breast somewhat tense, no lumps; protruding nipple, some nipple erosion, pinkish. Left breast: residual colostrum emission in the hospital, not performing stimulation; hypofunctional for lactopoiesis. Volume eliminated in the contralateral breast satisfies child's nutrition and allows storage. She underlines that she wants to schedule an appointment to talk about EBF.
Physical activities and exercise	Usual household activity does not impose mobility difficulties. Does not perform any physical exercise program.
Cognition and perception	Cognition and perception allow comprehensible communication. The puerperal woman is space- and time-oriented. She conveys awareness and a positive experience of motherhood.
Sleep and rest	The NB brought about certain alteration; she sleeps less than 8h/day. This does not value the disturbance, translating it by means of the expression “I'm a little tired”, but I rest when he lets me. She suggests trying to strike a balance in the rest-activity ratio.
Self-perception and self-concept	She has initiative to react to cancer mutilation, improving body image through a dermal strategy covering the scar. Self-perception towards development of her new roles. Investing in motherhood tasks, documenting herself, looking for health information and her own mother's *maternage* models. She recalls her self-image and body image with oscillations, reported in negative relational episodes. She suggests that those memories from her cancer history are actually made up for by happiness. Her self-image is cared for, according to the cultural gender standard. The maternal self-image suggests evolution, she attributed more value to self-concept, as she transmits through the commitment in EBF the appreciation of the quality of her milk, the NB's weight gain. There are still doubts and insecurities in the maternal roles.
Functions and relationships	She considers contact/relationship with her NB as her greatest value in life. Marital life seems to be preserved. Privileged relationship with her mother, which is the support replacing her episodically in care of the NB; her relationship is still tense with her father, due to his previous disagreement regarding the option of his daughter becoming a mother; considerable improvement and more friendly relationship postpartum.
Sexuality and reproduction	She self-describes as a person with a satisfactory sexuality; she found positive response mechanisms in social and intimate relations with her partner.
Facing and tolerating stress	She suggests being equipped with coping mechanisms to face difficulties. She focuses on obtaining adequate and positive answers. She suggests that she is living a happiness phase.
Values and beliefs	She emphasizes the value of motherhood and family.

On the 10^th^ postpartum day, data collection was performed through physical examination, consultation of the Nursing Process and behavioural observation of the dyad in breastfeeding instances. Given that breastfeeding suggested being the major concern, a script for data collection was prepared (Table 2). Through a semi-structured interview with audio-recording, guided by topics and indicators.^15^

**Table 2:**
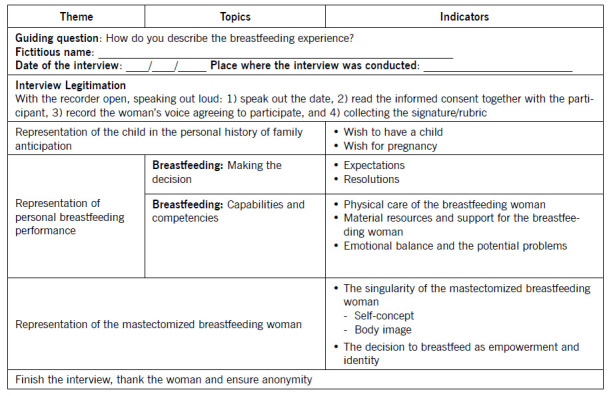
Interview script

The case was assessed in the light of the NANDA taxonomy,^16^ objectives were defined^17^, as well as interventions^18^ evaluating the results.

The ethical principles were preserved, ensuring confidentiality and anonymity. Written permission was obtained through signature of the informed consent from, according to the Convention of Oviedo (e.g., Resolution of the Republican Assembly. Diário da República No. 2/2001, Series I-A of 2001-01-03).

## Results

### Description of the clinical case

The case study reports a female person, 36 years old, Caucasian, married, with 12^th^ grade as academic qualifications. 

Health history. Left breast cancer history 8 years ago. Tumorectomy in the left supra-external quadrant with axillary emptying, G2 p T1c N1 (1/18gg) MO, RE10% RP10% Her2 Positive. She underwent 8 chemotherapy cycles and 25+7 radiotherapy sessions. Trastuzumab therapy for 1 year and Tamoxifen for 4 years. 

Obstetric history. Obstetric index (0;0;0;0). Unexpected pregnancy for the time, but wanted. In the presence of a positive pregnancy test, she suspended anxiolytic and antidepressant medications on her own initiative. She was monitored in PHC (8 appointments) and followed-up in oncology consultations. Evolution with no complications. She attended the Birth Preparation Program (*Programa de Preparação para o Nascimento*, PPPN). Caesarean section at 38W+4d, due to non-tranquilizing foetal status at 24h of labour, with 7 cm cervicometry. The first contact with the newborn (NB) was in the anaesthetic recovery room and the first breastfeeding instance was at 4 hours of life. Hospital discharge at 72 hours. NB data: APGAR 10/10, length 48.5 cm (percentile 15), weight 2,935 grams (percentile 12). 

Postpartum evaluation at day 10. Some assertions stand out in the interview: a) The wish to have a child/get pregnant: *"a son, I always wanted, even as a child... in games... He came a few months earlier, but I was counting on him"*; b) Capabilities and competence to Breastfeed: *“I really wanted to come and talk to the nurse, because during the PPPN, she said very important things to me (..) when she talked about breastfeeding…. it made me feel strong and able to decide what I wanted to do”*; c) Singularity of the mastectomized woman: *“I had to postpone the pregnancy because of this breast problem (…) time has passed… I need help…. to know how to deal with it in this issue of breastfeeding”*; d) Vacillating in the decision: *"I can breastfeed... I'm afraid it's not enough because it's from one breast, but I really want to... I give him what I have;* e) Empowerment: *(..) my family doesn't agree (…) but even if everyone says no, I want to give….at least try (…) I only stop it if it’s not enough for the baby (…) it's my body that's like this, which is like this now, but I can do it (…) the nurse realizes (…) I just need some help”.*

**Figure 1:**
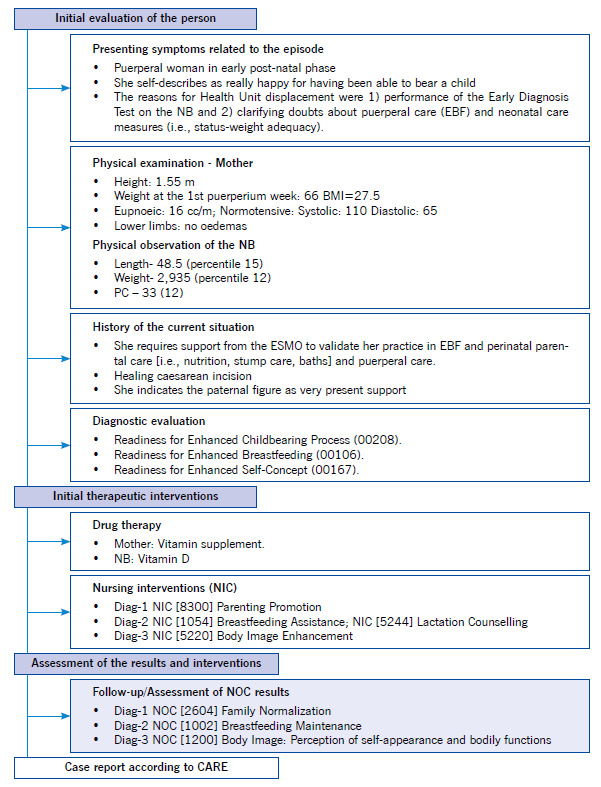
Flowchart

According the CARE guidelines, the following flowchart (Figure 1) showing the clinical aspects is presented.

Three Nursing diagnoses were defined: 1) Domain 8 - Sexuality. Diagnosis [00208] Readiness for Enhanced Childbearing Process; 2) Domain 2 - Nutrition. Diagnosis [00106] Readiness for Enhanced Breastfeeding; and 3) Domain 6 - Self-perception. Diagnosis [00167] Readiness for Enhanced Self-Concept. The clinical case was assessed through the NANDA-I domains^16^, and the Nursing Outcomes Classification^17^ and Nursing Interventions Classification (NIC)^18^ nomenclatures were used according to Table 3. 

The interventions performed (NIC) were assessed on the 10^th^ postpartum day and evaluated approximately two weeks later (Table 4). The evolution is recorded according to the NOC indicators.^17^

**Table 3:**
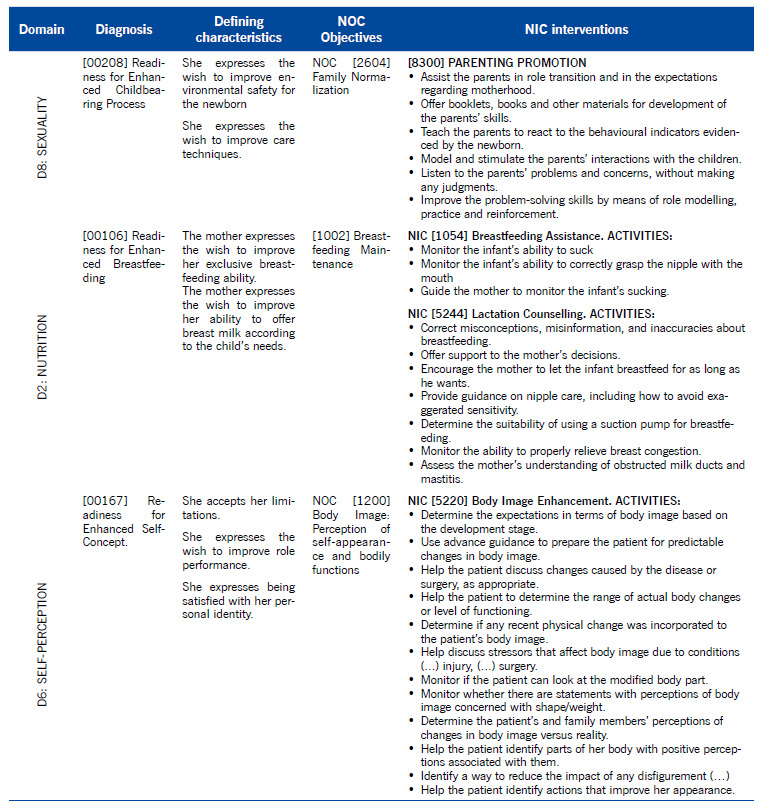
Diagnoses and interventions

**Table 4:** Assessment and evaluation of indicators

NOC	Indicators*	Initial evaluation	Final evaluation	
D8: SEXUALITY. [00208] Diagnosis: Readiness for Enhanced Childbearing Process [after birth]				
NOC 2604	[260408] Satisfies the developmental needs of family members	3 Sometimes shown	4 Frequently shown	+
	[260420] Maintenance of normal member expectations	3 Sometimes shown	4 Frequently shown	+
	Subtotal	6	8	
D2: NUTRITION. [00106] Diagnosis: Readiness for Enhanced Breastfeeding: Pattern of offering milk to the infant or young child directly from the breasts that can be improved				
NOC 1002	[100201] Infant growth within normal parameters	2 Slightly adequate	4 Substantially adequate	+
	[100205] Mother's ability to safely collect and store breast milk	1 Not adequate	5 Totally adequate	+
	[100207] Ability to safely defrost and gradually warm BM	1 Not adequate	4 Substantially adequate	+
	[1002018] Breast sensitivity prevention techniques	1 Not adequate	4 Substantially adequate	+
	[100208] Recognition of signs of reduced milk supply	2 Slightly adequate	4 Substantially adequate	+
	[100219] Recognition of signs of duct obstruction	1 Not adequate	3 Moderately suitable	+
	[100220] Recognition of signs of mastitis	1 Not adequate	3 Moderately suitable	+
	[100221] Awareness that breastfeeding can go beyond lactation	3 Moderately suitable	4 Substantially adequate	+
	[100222] She perceives family support for breastfeeding	2 Slightly adequate	3 Moderately suitable	+
	[100204] Knowledge about the benefits of maintaining BF	3 Moderately suitable	4 Substantially adequate	+
	Subtotal	17	38	
D6: Self-perception. [00167] Readiness for Enhanced Self-Concept. Pattern of perceptions or ideas about herself that can be improved				
NOC 1200	[120001] Self-portrait	4 Frequently positive	5 Consistent/Positive	+
	[120002] Coherence between the reality of the body, the ideal of the body and the presentation of the body	4 Frequently positive	5 Consistent/Positive	+
	[120003] Description of the affected body part	3 Sometimes positive	4 Frequently positive	+
	[120016] Attitude towards touching the affected body part	4 Frequently positive	5 Consistent/Positive	+
	[120017] Attitude towards using strategies to improve appearance	2 Rarely positive	5 Consistent/Positive	+
	[120005] Satisfaction with body appearance	3 Sometimes positive	5 Consistent/Positive	+
	[120018] Attitude regarding strategies to improve functions	3 Sometimes positive	4 Frequently positive	+
	[120006] Satisfaction with bodily function	4 Frequently positive	5 Consistent/Positive	+
	[120007] Adaptation to changes in physical appearance	2 Rarely positive	4 Frequently positive	+
	[120008] Adaptation to changes in bodily functions	2 Rarely positive	4 Frequently positive	+
	[120013] Adaptation to changes in the body resulting from the injury	3 Sometimes positive	5 Consistent/Positive	+
	[120014] Adaptation to body changes resulting from the surgery	5 Consistent/Positive	5 Consistent/Positive	=
	Subtotal	39	56	
Total		(6+17+39)=62	(8+38+56)=102	

## Discussion

Knowledge of real reports in the literature can facilitate the analysis of situations that arise in the clinic.^19^ Case studies or clinical cases have a didactic function, transmitting knowledge about the uniqueness of situations in a profound way, which in some aspects can be applied to others.^14^ Despite being classified as level of evidence 5, it is simultaneously an exercise in which the theory-practice-research relationship is clear.^20^ In the current case, the occurrence of oncological disease prior to pregnancy draws the attention to the EESMO responsibilities in the protection and prevention against breast and gynaecological cancer and for early diagnosis, according to recognized competence.^21^ Prevention is within the reach of EESMOs, as they carry out clinical screening programs and health education actions and can act on women of different ages. 

### Discussion about the NANDA-I diagnostics

*[00208] Readiness for Enhanced Childbearing Process*. The transition to parenting requires adapting to new roles and responsibilities. Childbearing processes are demanding for parental figures who, due to the absence of family care models, seek the support of EESMO professionals. In the puerperium phase, at the time of the Early Diagnosis (foot test) in the home visit, in the PHC breastfeeding corners or in the vaccination episodes, among others, it is nurses that identify the cases of women in crisis and sometimes manage to avoid or at least postpone EBF abandonment. In the current study, identification of the situation, the resources and guidelines offered by the EESMO through the NIC and NOC suggest that having responded to the puerperal woman's needs. The EESMOs have and advocate a professional profile of care guardians, in favour of expanding families,^22^ and their clinical potential may also be reflected in PHC.^23^ Some authors consider that these professionals even master an exercise space, the so-called *birth territory.*^24^ Attitudes of active listening and non-judgment and also the concrete problem-solving aspects facilitate improving care techniques and the feeling of environmental safety. The final evaluation suggests that the participant recognizes that the family has entered its normalization at this development phase.

*[00106] Readiness for Enhanced Breastfeeding*. In women with serious conditions prior to pregnancy and who opt for EBF, sometimes difficult situations arise that justify individualized counselling that conveys security^25^ and may facilitate decision making. In the current case, counselling was present both in the initial assessment, with observation and guidance on the breastfeeding instance, and in monitoring of the texture of the functioning contralateral breast and the non-functioning breast. Other guidelines that were offered on the 10^th^ day allowed for absence of engorgement, duct blockage or severe nipple erosion. On the other hand, the care measures also provided attention to the non-functioning breast, as the puerperal hormonal situation is different and implies surveillance of the breast-nipple unit.^6^ This was important since, in most puerperal women and even without any pathology, engorgement, blockage of the milk ducts and nipple pain are the most frequent reasons for EBF abandonment. EBF is an option generally rejected by women with chronic diseases.^3^ It is even discouraged by health professionals when there are pathologies sensitive to the hormonal environment of the puerperium.^26^ The same fear arises when the disease is debilitating and vertically transmissible, there is proximity contagion or even for fear that the drugs pass into human milk.^3^ In the case of the current participant, although her cancer history was close, EBF was her own choice, with a determined attitude but initially insecure due to her doubt regarding meeting the NB's nutritional needs. Self-efficacy in EBF is perhaps the greatest doubt for mothers who breastfeed for the first time; however, a number of studies reveal that positive attitudes from professionals, family and social support contribute to greater maternal confidence and security.^27^

*[00167] Readiness for Enhanced Self-Concept*. According to the final evaluation, the care offered responded to the puerperal woman's needs, as expected from the EESMO knowledge. In NIC [5220] Body Image Enhancement, the EESMO guidelines sought to respond by valuing the puerperal woman and jointly discovering aspects to self-value. However, the NANDA-I taxonomy seems to be lacking in the assessment of developmental, non-pathological self-image in the puerperium. An example is “Using advance guidance to prepare the patient for predictable changes in body image” from the NIC, which suggests not contemplating changes in body image in the pregnancy-puerperal cycle, as expected and desired by the woman and by the EESMO. The same happens in “Helping the patient to determine the scope of the real changes in the body or functioning level” from the NIC since the changes, namely lactation, are not a pathological process. Also in “Monitor whether there are statements that identify perceptions of body image concerned with body shape and weight” from the NIC, in fact in pregnancy and the puerperium, changes in weight and volume are expected, desired and valued in the opposite direction to the feminine thinness stereotypes. Also “Determine the perceptions of the patient and family about changes in body image *versus* reality” from the NIC since, for the pregnant/postpartum woman and her family, the change in body image has, above all, a positive meaning of reproductive capacity and femininity. 

The final evaluation suggests that the participant's care demand and what was offered by the EESMO contributed to success. In fact, in some evaluation items, the participant had already reached a state of acceptance (for example: [120014] Adaptation to body changes resulting from surgery On the other hand, among others, items [120007] Adaptation to changes in physical appearance and [120008] Adaptation to changes in bodily functions showed an increase in the last evaluation. This suggests that appreciation of the female body that nourishes the child seems to have overlapped with the bodily losses resulting from breast surgery. On the other hand, items [120017] Attitude towards using strategies to improve appearance and [120005] Satisfaction with body appearance suggest that, in the roles played by the EESMO and the participant, a supportive bond was reproduced, which replicates the ancestral alliance between women. In facts related to birth, this phenomenon is a trait of the species that perpetuates care in the face of human alterity and the altricial nature of the offspring. 

Implications *for the professional practice and health policies.* An EESMO is a health technician who clearly and quickly understands the pregnancy and delivery experience (re)told by puerperal women. Thus, from this point of view, the presence of more EESMOs in PHC is advocated. This contributes to puerperal women's quality of life and renders them loyal to the surveillance of their own health and that of their newborns, reducing expenses, severity of the situations and late care demand. Recognizing the EESMO roles and facilitating innovative projects in their space, in *Birth Territory*, can improve the immediate and long-term health of a woman and her child. It will be important for health policies to value EBF and a robust reference to breastfeeding in the National Health Plan is extremely desirable, devised for the next decade. 

## Limitations

The case study method precludes generalization of the results. Despite the prototype inherent to the diagnostic model, it was guided by the participant's unique needs.

## Conclusion

Motherhood after cancer is a challenge. There are fears for the present, in the uncertainty of the current capabilities, and fears for the future, in doubt whether there will be time for answers. The diagnoses identified are common in primiparous women when faced with the EBF decision, as well as the need to prove the child's status-weight development. It is important to strengthen maternal self-image, valuing any woman who breastfeeds, particularly when she has undergone a mastectomy. Using the NANDA-I taxonomy revealed difficulties in the [00167] Readiness for Enhanced Self-Concept diagnosis, given that maternal self-image entered into conflict with the body image of the mutilated body and did not fully respond to the puerperal changes. The diagnoses reveal a social environment with a weak representation of *Maternage*, reflecting a country where generation replacement has not occurred since the 1980s. Maternal care measures are currently learned in a medicalized way, through the PPPN, and developed by health professionals. In health education for women in the puerperal phase, active listening and genuine interest in the person requesting support are fundamental. Devising a set of solutions applied to the characteristics of each case can translate into more rewarding motherhood experiences.
